# Engineered 3D-Printed Poly(lactic acid) and Acrylonitrile Butadiene Styrene (ABS) Membranes’ Caster for Preparation of Chitosan-Based Liquid Membranes: A Short Communication

**DOI:** 10.1021/acsomega.6c04590

**Published:** 2026-07-15

**Authors:** Ahmad Fikri Adam, Ayo Olasupo, Matthew Y. Lui, Kumar Sudesh, Faiz Bukhari Mohd Suah

**Affiliations:** † Green Analytical Chemistry Laboratory, School of Chemical Sciences, 26689 Universiti Sains Malaysia, 11800 Minden, Pulau Pinang, Malaysia; ‡ Wonderful Institute for Sustainable Engineering, Chemical and Petroleum Engineering, 4202 University of Kansas, 1530, W. 15th Street, Lawrence, Kansas 66045, United States; § Department of Chemistry, 26679 Hong Kong Baptist University, Waterloo Road, Kowloon Tong, Kowloon, HKSAR 999077, P. R. China; ∥ Ecobiomaterial Laboratory, School of Biological Sciences, Universiti Sains Malaysia, 11800 Minden, Pulau Pinang Malaysia

## Abstract

Chitosan membranes have attracted growing interest for separation and sensing applications owing to their biodegradability, chemical functionality, and film-forming ability. However, strong adhesion to the casting surface makes conventional Petri dish fabrication challenging. In this study, a three-dimensional (3D) membrane caster was fabricated via fused deposition modeling (FDM) with a removable Teflon base sheet, enabling reliable casting and easy detachment of membranes. The caster is reusable, customizable in size and geometry, and compatible with both of the conventional filaments, polylactic acid (PLA) and acrylonitrile butadiene styrene (ABS) filaments, without interfering with membrane integrity. Characterization by fourier transform infrared spectroscopy (FTIR), scanning electron microscopy (SEM), and EDX confirmed comparable results between membranes cast on the 3D caster and a conventional glass Petri dish. Water uptake (∼93–94%) and thickness distribution (∼35–43 μm) were consistent across all casting platforms, indicating negligible differences in swelling behavior and uniformity. This scalable approach is readily adaptable to other membrane types and offers a practical fabrication model for liquid membranes (LMs) and polymer inclusion membranes (PIMs) in future separation systems.

## Graphical abstract

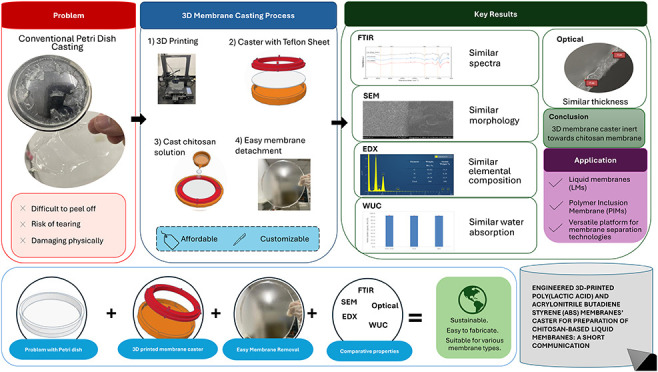

## 1 Introduction

Chitosan is a natural polysaccharide that is derived from the deacetylation of chitin, which has attracted a lot of attention as a membrane material in the separation system due to its biodegradability and chemically modifiable properties for a certain function.^1,2^ The morphology and performance of chitosan membranes are, however, strongly influenced by the fabrication method, casting substrate, and drying conditions.^3,4^ Conventional solvent-casting using a Petri dish remains the most common approach, yet it often results in nonuniform thickness, edge defects, and difficulties in membrane removal from the substrate surface.^5^ Nonetheless, the casting method using a Petri dish has difficulty producing a uniform, easy-to-peel product.

Currently, additive manufacturing technologies have gained considerable interest in membrane technology. Among these technologies, fused deposition modeling (FDM), one of a material extrusion (MEX) technique, has been increasingly integrated into membrane fabrication due to its low cost, customizable geometries, biodegradability, and reproducibility.^6–8^ The potential of making a 3D caster is demonstrated by a past study in which 3D printing enabled the fabrication of ultrathin membranes and module components, such as feed spacers, supports, and entire modules, with complex morphology and functionality beyond what conventional casting allows.^9^ One study has combined printed separation and integrated membranes, achieving enhanced design versatility and reducing assembly steps.^10^ Innovations include iso-porous membranes fabricated directly via 3D printing, with feature sizes down to ∼7 μm, demonstrating the potential for high-resolution membrane construction.^11^ Some works apply FDM to create pressure-driven polymer membranes by blending PLA with porogens and leaching steps.^12^ 3D printing enables the layer-by-layer fabrication of membranes with intricate geometries and hierarchical structures. Techniques such as masked stereolithography and photo polymerization-induced phase separation enable the fabrication of membranes with customized pore sizes and porosity, greatly enhancing the effectiveness and precision of filtration.^13^

Therefore, the 3D casting can replace the Petri dish, offering lower cost and greater reproducibility. To obtain a perfect membrane, Teflon (PTFE) sheets were used as the base in the 3D membrane caster due to their high chemical resistance and low surface energy, making them a nonreactive, nonstick substrate.^14^ Furthermore, Teflon remains stable at high temperatures, maintaining flatness and supporting consistent membrane thickness.^15^ A recent study used PTFE as a support in harsh chemical and membrane separation settings, citing these advantages explicitly.^16^

Although 3D printing has been widely used in membrane science to produce membrane supports and functional structures, its application to the development of customized membrane casting platforms remains limited. There are relatively few reports focusing specifically on the use of 3D-printed holders or supports for casting membranes to reduce the risk of damaging the membrane’s physical structure and to customize different membrane shapes. In this study, a 3D-printed membrane caster with a detachable Teflon base is presented to address these challenges. Casters made from polylactic acid (PLA) and acrylonitrile butadiene styrene (ABS) were compared to assess their effects on membrane uniformity, ease of removal, and durability. The widespread use of PLA in drug delivery systems and biomedical devices is due to its biodegradability and ease of processing, whereas ABS is used in laboratory equipment for its toughness and thermal resistance, making them the most widely used thermoplastic materials in FDM.^17,18^ Various thermoplastic polymer systems have been investigated for 3D printing applications, including PLA/PBAT, PLA/TPU, and PVC/PCL, due to their enhanced flexibility, toughness, and thermal properties.^19–22^ However, the requirements for the membrane casting device differ from those of functional structural components. The selection of PLA and ABS in this study because of their widespread availability, low cost, ease of printing, and suitability for producing reusable laboratory equipment.

While the present study focuses on chitosan membranes as a proof-of-concept system, the proposed 3D printed membrane caster may also be applicable to other solution-cast membrane systems. In particular, the removable Teflon-supported design may be beneficial for liquid membranes (LMs) and polymer inclusion membranes (PIMs), in which removing membranes from conventional casting substrates can be challenging. Therefore, the proposed platform may offer a versatile, reusable casting approach for future membrane fabrication studies.

## 2 Materials and Methods

Chitosan (200–600 mPa.s with degree of deacetylation ≥ 80.0%) was obtained from Tokyo Chemical Industry (Tokyo, Japan), and glacial acetic acid (purity ≥ 99.9%) was obtained from Sigma-Aldrich (St. Louis, MO). The polylactic acid (PLA) filament and acrylonitrile butadiene styrene (ABS) filament were obtained from 3DExpress (Kuala Lumpur, Malaysia). The Fused Deposition Modeling (FDM) 3D printer used was Creality (CR-6 SE) (Figure 1). The Teflon sheet was obtained from Tool Solid (Pulau Pinang, Malaysia).

**Figure 1:**
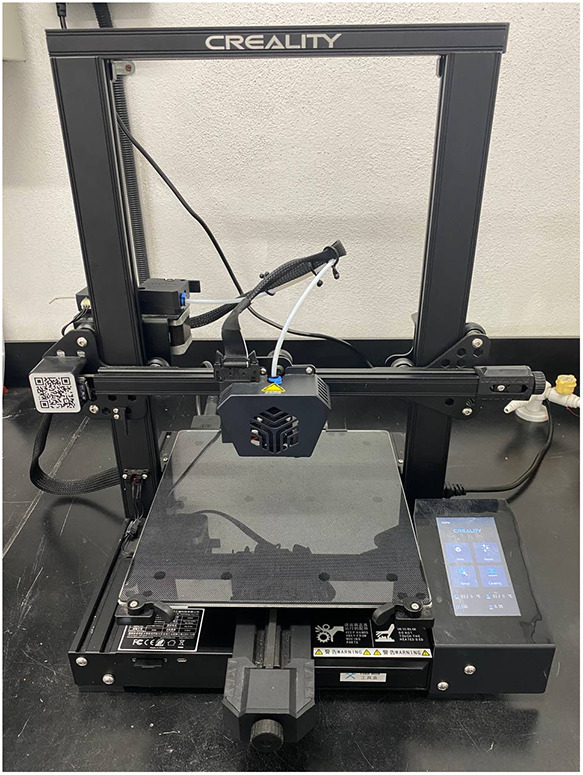
FDM Printer, Creality CR-6 SE.

The 3D membrane caster model was designed in TinkerCad, an online platform for designing 3D models (Figure 2). The model was saved as an STL file and sliced in Creality Slicer 4.8.2 using the appropriate parameters (Table 1). The 3D membrane caster was printed on a Creality CR-6 SE and took about 5 h to complete. The design parameters are listed in the Supporting Information (Table S1). The Teflon sheets were cut to 90 × 90 mm^2^ and used as a base cover for the 3D membrane caster.

**Figure 2:**
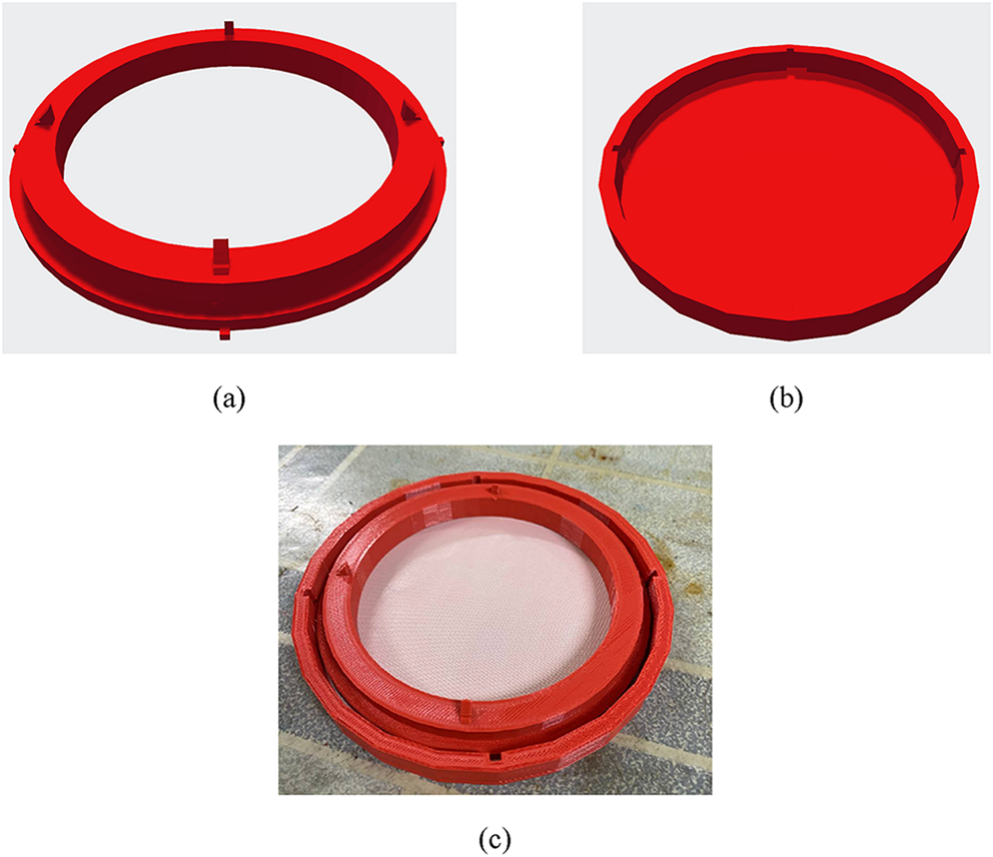
(a) The top holder, (b) the base of the 3D caster, and (c) printed 3D caster with a Teflon sheet as a base.

**Table 1:** Parameters of the 3D Casting in Creality Slicer 4.8.2

parameters	size
dimension	90 × 90 × 10 mm^3^
wall thickness of the membrane caster design	10 mm
wall line count	2
infill density	35%
infill line distance	1.5 mm
infill pattern	gyroid
layer height	0.2 mm
printing speed	85 mm/s
nozzle	0.2 mm
nozzle temperature	PLA: 220 °C ABS: 250 °C
bed temperature	PLA: 70 °C ABS: 80 °C

For preparing the chitosan membrane, 200 mg of chitosan and 20 mL of 1% v/v wt % acetic acid were added to a 250 mL beaker. The mixture was stirred for 5 h to ensure the chitosan was fully dissolved. The mixture was poured into the 3D membrane caster holder and dried in a controlled fume hood (25 ± 2 °C) for 72 h. Subsequently, the membrane was removed from the 3D membrane caster and washed with distilled water. The membrane became usable after drying in an oven for 2 h.

The functional groups of the chitosan membranes were analyzed using Fourier Transform Infrared-Attenuated Total Reflectance (FTIR-ATR, PerkinElmer Frontier IR Model SPOTLIGHT 200). The spectra were recorded in the range of 4000–400 cm^–1^ with a resolution of 4 cm^–1^ and over 32 scans. The membranes were dried at room temperature before analysis to remove moisture-related interference. The obtained spectra were used to confirm the characteristic peaks of chitosan and were compared with those reported in similar studies in the literature.

The morphology of the membranes was characterized using a field-emission scanning electron microscope (FE-SEM, Hitachi-Regulus 8240). Small pieces of membrane were mounted on platinum stubs and coated with gold to prevent charging. Surface images were captured at different magnifications to compare the morphology of membranes cast in Petri dishes versus those cast using the 3D caster. The elemental composition of the membranes was analyzed using Energy Dispersive X-ray Analysis (EDX). The spectra were collected to identify the primary constituents of chitosan: carbon (C), nitrogen (N), and oxygen (O).

The overall surface uniformity and thickness distribution of the membranes were examined using an optical microscope (VHX-XF Series) under reflected light. Images were captured at different points across the membrane surface. The membrane thickness was determined using the microscope’s calibrated micrometer scale provided in the system. The water absorption of the chitosan membranes was determined by weighing a dry membrane (*W*_b_) and then immersing it (2 × 2 cm^2^) into ultrapure water for 24 h at room temperature to obtain the wet membrane (*W*_a_), The water uptake capacity (WUC) was calculated using methods reported by Khodami et al. (2020)^23^

1.0 $$\mathrm{WUC}=\frac{W_{a}-W_{b}}{W_{b}}\times 100\%$$

## 3 Results and Discussion

After 3 days of solvent evaporation, the chitosan membrane was peeled off by twisting the 3D caster, and the chitosan membrane was obtained as shown in Figure 3.

**Figure 3:**
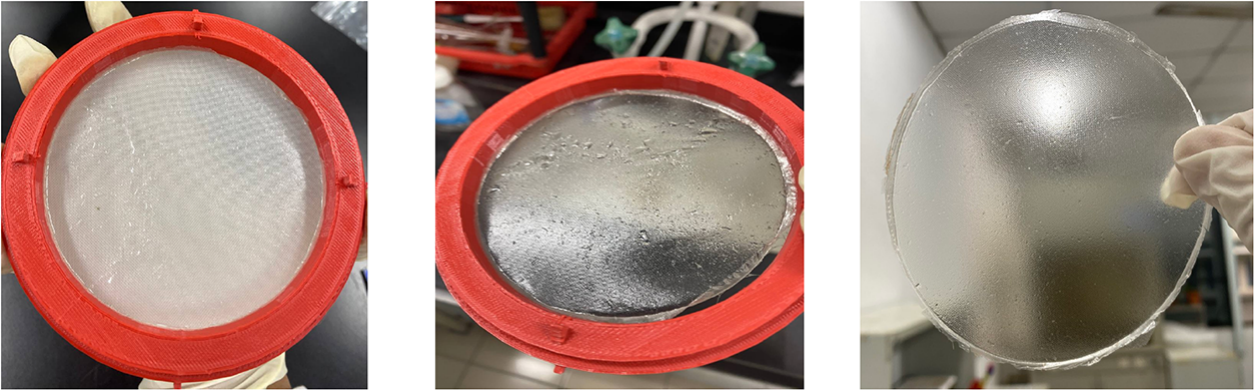
Chitosan membrane peeled off from the 3D caster.

The FTIR spectra of chitosan membranes fabricated with various casting support (Petri dish, PLA, and ABS) showed a similar characteristic absorption bands at approximately 3400 cm^–1^ (O–H and N–H stretching), 2927 cm^–1^ (C–H stretching), 1590 cm^–1^ (N–H bending of amine groups), and 1060 cm^–1^ (C–O–C stretching of the glycosidic linkage), which are indicative of chitosan (Figure 4). Based on previous studies, these bands indicate that the polysaccharide structure is present in all samples.^24,25^ No additional peaks were observed in membranes cast on the PLA or ABS 3D caster, indicating that the 3D caster did not interact with chitosan. However, there are slight differences in the intensity and breadth of the 3400 cm^–1^ and 1590 cm^–1^ bands that may be due to small changes in hydrogen bonding or water retention caused by variations in surface energy of the casting materials.^26^ Overall, the FTIR analysis indicates that both 3D casting materials are chemically inert toward the chitosan.

**Figure 4:**
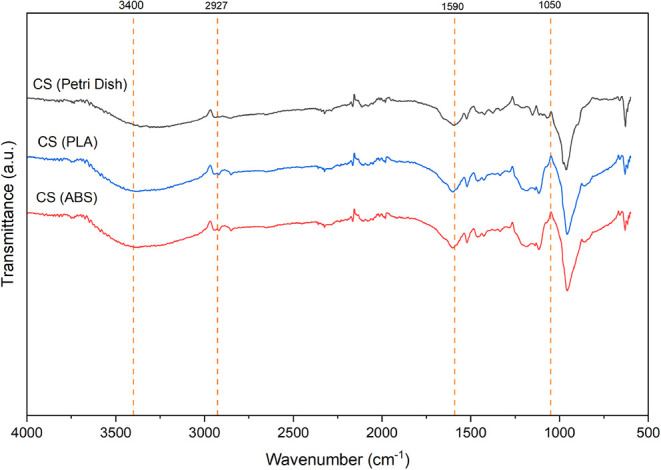
FTIR spectra of pure chitosan membranes by the Petri dish caster, PLA-based, and ABS-based membrane caster.

However, slight variations in the intensity and broadness of the 3400 cm^–1^ and 1590 cm^–1^ bands suggest that minor changes in hydrogen bonding or water retention related to surface energy differences of the casting materials.^26^ Overall, the FTIR analysis demonstrates that both PLA and ABS 3D casters are chemically inert toward chitosan, confirming their suitability for use in membrane fabrication.

From Figure 5(a,b,c), the surfaces of all membranes were relatively continuous and defect-free. The membrane prepared by the conventional Petri dish caster had a visually more uniform surface, whereas the membranes prepared by the 3D membrane casters showed minor surface irregularities that may be associated with the differences in the base surface. However, the PLA and ABS 3D casters yielded membranes with comparable morphology features. No obvious cracks, large pores or phase separation were observed in any of the membranes. The observed morphology is consistent with that of the previously reported chitosan membranes.^1,27^ In general, SEM images indicate that the 3D membrane caster did not cause significant morphological defects in the produced membranes.

**Figure 5:**
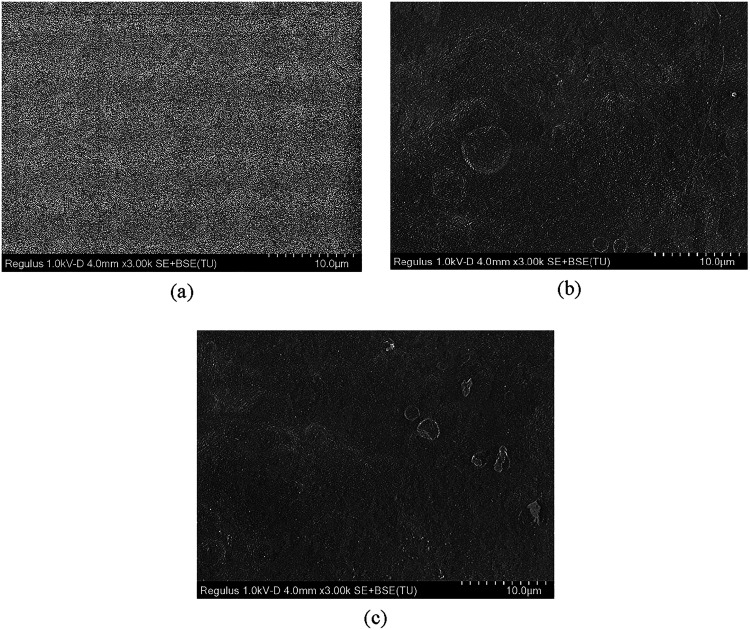
(a) SEM images of the chitosan membrane prepared by the Petri dish, (b) by PLA-based membrane caster, and (c) ABS-based membrane caster. Images were acquired at an accelerating voltage of 10 kV.

As shown in Figure 6, the elemental compositions of all the membranes from the caster were similar: carbon (C), oxygen (O), and nitrogen (N). These elements are the major constituents, consistent with the chemical formulation of chitosan reported in previous studies.^28,29^ The atomic weight % for all the membranes produced by different casters was 71.3%, 5.20%, and 23.5%, respectively. The carbon and oxygen signals originated from the polysaccharide backbone, hydroxyl, and ester groups, whereas the nitrogen signal reflected the amino groups of chitosan. PLA is composed of C, O and hydrogen (H), whereas ABS is composed of C, H, and H based on the functional groups present in the material. Since EDX cannot detect H due to its low atomic number, it was expected to show an increase in the abundance of elements such as C, O, and N in the membranes from PLA and ABS casters. However, there were no abnormal peaks in the EDX spectra, and since they yielded similar results to the Petri dish caster, this indicates that the membrane was inert toward the 3D membrane caster.

**Figure 6:**
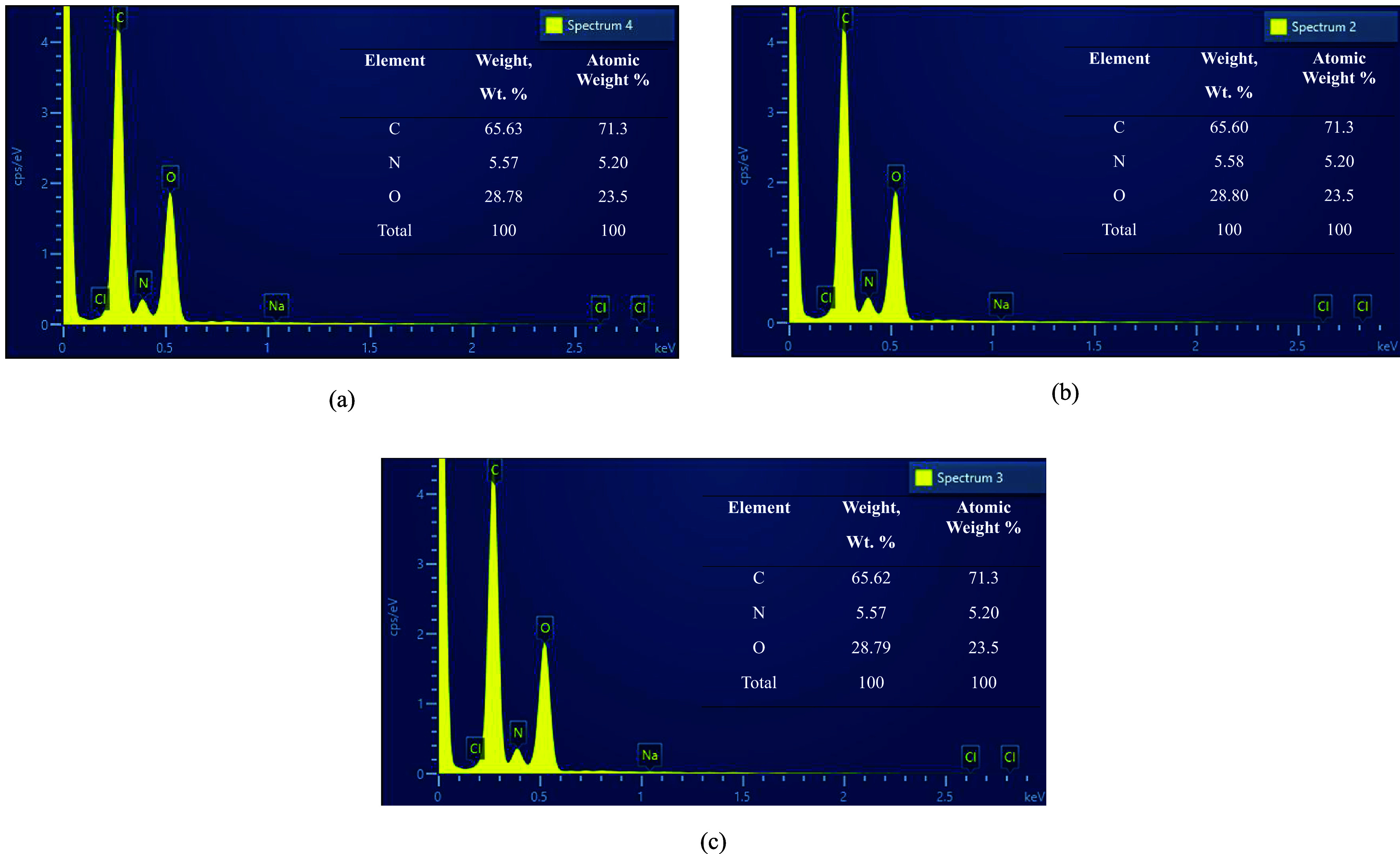
EDX spectra of the chitosan membranes prepared using (a) Petri dish casting, (b) PLA-based membrane caster, and (c) ABS-based membrane caster.

The SEM and EDX results show that the 3D casting method produces a membrane with a surface morphology and chemical composition comparable to those of the membrane produced in a Petri dish. The resemblance between the Petri dish and 3D casting membranes confirms that 3D casting is a reliable alternative for membrane fabrication.

The average thickness of chitosan membranes prepared by Petri dish casting was 35.2 μm (center) and 38.0 μm (edge), while the average thickness of membranes prepared using the caster based on PLA was 41.2 μm (center) and 43.4 μm (edge), as shown in Table 2. Similarly, the ABS-based caster yielded membranes with average thicknesses of 41.0 μm (center) and 43.2 μm (edge). The measured thickness values for all the fabrication methods lie within a narrow range and are comparable to previous studies on solvent-cast chitosan membranes, which report 36–43 μm for ZIF-8/chitosan composite active layers, and align with the broader range of 18–110 μm depending on formulation and processing.^30^ Statistical analysis, including standard deviation, minimum (Min), maximum (Max) and coefficient of variation (CV), was performed for detailed analysis of the distribution of thickness (Table 2). The CV values for all the samples were between 2% and 4%, indicating that the thickness in both the center and edge areas had low variability. The CV values for the Petri dish membranes were 2.24% (center) and 3.04% (edge), and for the PLA- and ABS-cast membranes, they ranged from 1.91% to 3.29%, respectively. These results indicate that the 3D-printed casting platforms do not introduce significant additional variation in thickness compared to conventional casting. Even though there were small differences in the absolute thickness between the Petri dish and the 3D-printed casters, all membranes were in a similar thickness range (around 35–43 μm), indicating that the casting platform does not significantly influence the final membrane thickness. Furthermore, the application of a Teflon-supported base helped remove the membrane without visible deformation and allowed handling without affecting its structure. The cross-sectional thickness of the membrane produced by these casters is shown in Figure 7.

**Figure 7:**
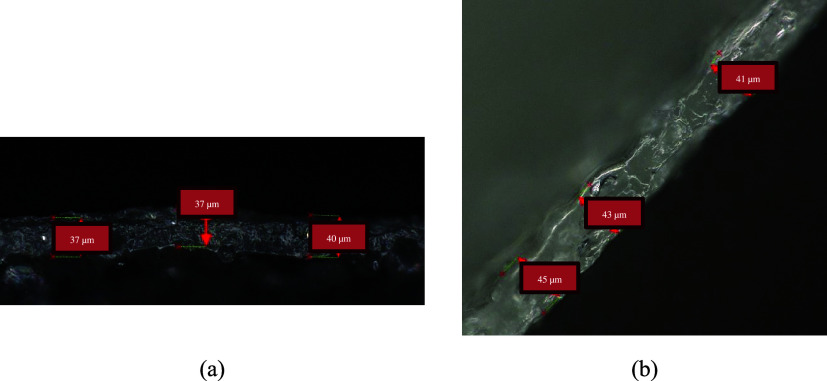
Representative cross-sectional thickness measurements of chitosan membranes produced by (a) Petri dish casting and (b) 3D printed membrane casting. Quantitative thickness statistics are summarized in Table 2.

**Table 2:** Thickness Distribution of Chitosan Membranes Fabricated from Petri Dish Casting and 3D Printed Membrane Casters^a^

	petri Dish		PLA		ABS	
membrane caster	center	edge	center	edge	center	edge
mean (μm)	35.2	38.0	41.2	43.4	41.0	43.2
min (μm)	34.0	37.0	40.0	41.0	40.0	41.0
max (μm)	36.0	40.0	42.0	45.0	42.0	45.0
SD (μm)	0.79	1.15	0.79	1.14	0.94	1.14
CV (%)	2.24	3.04	1.91	2.63	2.30	2.64
E-C difference	+ 2.8 μm		+ 2.2 μm		+ 2.2 μm	

a SD: standard deviation, CV: coefficient variant, E-C: edge-center.

As for the water uptake capacity of the chitosan membranes, it was around 93.0–94.0% with insignificant variation across different casting platforms (Figure 8). This resulted in the retention of chitosan’s hydrophilic character regardless of the fabrication method. The obtained values agree with the water uptake behavior of uncoated chitosan membranes reported previously, which also show water absorption in the range of about 90–100%.^31^ The agreement further supports the conclusion that the proposed 3D-printed membrane caster does not alter the intrinsic swelling behavior of chitosan membranes.

**Figure 8:**
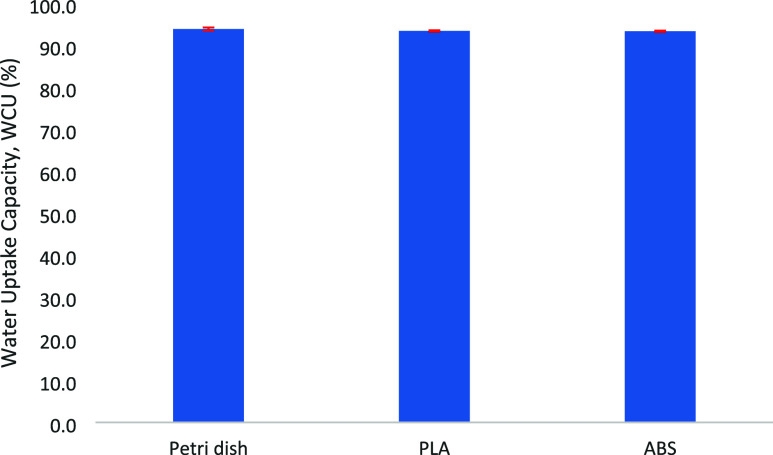
Comparison of WUC of chitosan membranes produced by different casting platforms. Data are presented as mean ± standard deviation (*n* = 5).

## 4 Conclusion

In this research, pure chitosan membranes were effectively produced using conventional Petri dish casting and 3D-printed casting platforms (PLA and ABS), with a Teflon sheet as the base. The 3D casting method produces structurally intact chitosan membranes with thickness characteristics comparable to those of the conventional Petri dish, enabling easier membrane removal and reducing edge defects. FTIR analysis confirmed this, showing similar functional groups in the chitosan membrane across all casting methods. The morphological structure and thickness of the membrane were observed using SEM and optical microscopy, revealing that the membrane cast with the 3D caster was similar to that cast in a Petri dish. Furthermore, EDX analysis confirmed that the elemental composition of chitosan was identical across all membranes, indicating that the 3D caster materials did not affect the membrane composition. The WUC analysis showed that the chitosan membranes remained consistent across the different casting platforms, indicating that the 3D caster materials did not affect the membranes’ hydrophilic properties.

Overall, this study shows that the 3D casting method, whether using PLA or ABS, can be a practical and reproducible alternative to the conventional Petri dish casting method. The findings of this study open new avenues for the easy preparation of LMs and PIMs without the need to utilize the conventional Petri dish casting method, thereby easing the production of LMs and avoiding the difficulties of peeling them off. The design, thickness, and compositional variability of the membrane can be further studied using a 3D caster with different shapes.

## Supplementary Material





## Data Availability

The 3D design files for the 3D membrane caster in this study are in the Supporting Information file and also deposited in the Zenodo repository as STL files. The STL files are divided into two files, 3D Membrane Caster (TOP), and 3D Membrane Caster (BASE). These files can be accessed at DOI: 10.5281/zenodo.21096296

## Notes

Parameters in Creality Slicer 4.8.2 for each 3D membrane caster with PLA and ABS (Table S1) (PDF)
3D membrane caster (MP4)

## Abbreviations

ABS: acrylonitrile butadiene styrene
EDX: energy dispersive X-ray analysis
FE-SEM: field-emission scanning electron microscope
FDM: fused deposition modeling
FTIR-ATR: Fourier transform infrared-attenuated total reflectance
LM: liquid membrane
PLA: polylactic acid
PIM: polymer inclusion membrane.
