# Supplementary material for: Engineered 3D-Printed Poly(lactic acid) and Acrylonitrile Butadiene Styrene (ABS) Membranes’ Caster for Preparation of Chitosan-Based Liquid Membranes: A Short Communication
Source: ACS Omega. 2026 Jul 15;11(29):44294–300. doi: 10.1021/acsomega.6c04590 (PMC13425288; doi:10.1021/acsomega.6c04590)
Supplement: Supplementary file 1 [file ao6c04590_si_001.pdf]

ENGINEERED 3D-PRINTED POLY(LACTIC ACID) AND ACRYLONITRILE BUTADIENE STYRENE (ABS) MEMBRANES' CASTER FOR PREPARATION OF CHITOSAN-BASED LIQUID MEMBRANES: A SHORT COMMUNICATION

Electronic Supplementary Information

*Ahmad Fikri Adam[1], Ayo Olasupo[2], Matthew Y. Lui[3], Kumar Sudesh[4] and Faiz Bukhari Mohd Suah[1]**

[1]Green Analytical Chemistry Laboratory, School of Chemical Sciences, Universiti Sains Malaysia, 11800 Minden, Pulau Pinang, Malaysia, [2]Wonderful Institute for Sustainable Engineering, Chemical and Petroleum Engineering, University of Kansas, 1530, W. 15th Street, Lawrence, Kansas, 66045, United States, [3]Department of Chemistry, Hong Kong Baptist University, Waterloo Road, Kowloon Tong, HKSAR 999077, PR China, [4]Ecobiomaterial Laboratory, School of Biological Sciences, Universiti Sains Malaysia, 11800 Minden, Pulau Pinang, Malaysia.

Content

Table S1: Parameters in Creality Slicer 4.8.2 for each 3D membrane caster with PLA and ABS.

**Parameters**	**PLA**	**ABS**
Nozzle Temperature	220 ºC	250 ºC
Bed Temperature	70 ºC	80 ºC

DOI : https://doi.org/10.5281/zenodo.21096296

Model of 3D Printer : Creality CR-6(SE)

Size of Nozzle : 0.2 mm

Wall line count : 2

Infill density : 35%

Infill line distance : 1.5 mm

Infill pattern : Gyroid

Layer height : 0.2 mm
